# Structure Determination and Biochemical Characterization of a Putative HNH Endonuclease from *Geobacter metallireducens* GS-15

**DOI:** 10.1371/journal.pone.0072114

**Published:** 2013-09-06

**Authors:** Shuang-yong Xu, Alexandre P. Kuzin, Jayaraman Seetharaman, Alice Gutjahr, Siu-Hong Chan, Yang Chen, Rong Xiao, Thomas B. Acton, Gaetano T. Montelione, Liang Tong

**Affiliations:** 1 New England Biolabs, Inc. Research Department, Ipswich, Massachusetts, United States of America; 2 Department of Biological Sciences, Northeast Structural Genomics Consortium, Columbia University, New York, New York, United States of America; 3 Center for Advanced Biotechnology and Medicine, Department of Molecular Biology and Biochemistry, Rutgers University, Department of Biochemistry, Robert Wood Johnson Medical School, Northeast Structural Genomics Consortium, Piscataway, New Jersey, United States of America; NCI-Frederick, United States of America

## Abstract

The crystal structure of a putative HNH endonuclease, Gmet_0936 protein from *Geobacter metallireducens* GS-15, has been determined at 2.6 Å resolution using single-wavelength anomalous dispersion method. The structure contains a two-stranded anti-parallel β-sheet that are surrounded by two helices on each face, and reveals a Zn ion bound in each monomer, coordinated by residues Cys38, Cys41, Cys73, and Cys76, which likely plays an important structural role in stabilizing the overall conformation. Structural homologs of Gmet_0936 include Hpy99I endonuclease, phage T4 endonuclease VII, and other HNH endonucleases, with these enzymes sharing 15–20% amino acid sequence identity. An overlay of Gmet_0936 and Hpy99I structures shows that most of the secondary structure elements, catalytic residues as well as the zinc binding site (zinc ribbon) are conserved. However, Gmet_0936 lacks the N-terminal domain of Hpy99I, which mediates DNA binding as well as dimerization. Purified Gmet_0936 forms dimers in solution and a dimer of the protein is observed in the crystal, but with a different mode of dimerization as compared to Hpy99I. Gmet_0936 and its N77H variant show a weak DNA binding activity in a DNA mobility shift assay and a weak Mn^2+^-dependent nicking activity on supercoiled plasmids in low pH buffers. The preferred substrate appears to be acid and heat-treated DNA with AP sites, suggesting Gmet_0936 may be a DNA repair enzyme.

## Introduction

*Geobacter* bacteria grow under anaerobic conditions in soils and aquatic sediment. *Geobacter* species can oxidize organic compounds and metals, including iron and manganese in anaerobic environment leading to the production of electrons which can be harnessed in bioreactors [1]. Mixed microbial cultures can also generate hydrogen from consortium of *Geobeacter* species in a microbial electrolysis cell [2]. Biomass such as compost materials, plant biomass, human and animal solid waste can be converted into electricity or hydrogen by *Geobacter*’s metabolic activities. Thus, *Geobacter* bacteria are important target for study in both bio-remediation applications and development of renewable energy. *Geobacter metallireducens* GS-15 was first isolated from aquatic sediment [3]. The circular genome has been sequenced in the size of 3,997,420 bp, encoding ∼3,519 predicted gene products [4]. Being a bacterium growing in sediment, *G. metallireducens* GS-15 harbors restriction-modification (R-M) systems, presumably to fight off phage infections and invasion of foreign DNA. There are predicted restriction-modification (R-M) systems including a type IIS (GmeI, GGATC 4/5) and a type III (GmeII, TCCAGG 25/27), an orphan C5 methylase and an Mrr-like type IV restriction enzyme (see REBASE) [5], [6]. In addition, we found a putative HNH-family endonuclease, ORF number Gmet_0936, that contains the conserved catalytic residues His-Asn-Asn (HNN), and a zinc binding domain (zinc ribbon) consisting of the conserved motif of [CxxC]_2_. Companion DNA methyltransferase (MTase) is not found adjacent to Gmet_0936 so it is unlikely that this putative HNH endonuclease is part of an active R-M system. Gme_0936, however, is located in proximity to Gmet_0935, a predicted RNase HI homolog, and to Gmet_0939, a gene predicted to be involved in repair of stalled DNA replication fork.

The HNH endonuclease catalytic domain with a zinc ribbon (ββα-Me fold) has been found in type II restriction endonucleases (e.g. Hpy99I and PacI), non-specific endonucleases (Colicins), DNA repair enzymes (MutS), and homing endonucleases (e.g. I-HmuI). Therefore, it is important to understand the structure and biochemistry of this group of enzymes. The goal of this work is to solve the crystal structure of Gmet_0936 protein and to study the enzymatic activity of this putative endonuclease. Here we report the expression, purification, and structural determination of Gmet_0936 at 2.6 Å resolution. In addition, we report a weak DNA binding activity of Gmet_0936 protein and the DNA nicking activity of the wt enzyme and its variant N77H. The possible biological function of Gmet_0936 (Gme HNH endonuclease or Gme HNHE) is discussed.

## Materials and Methods

### Protein Expression and Purification for Crystallography

The full-length gene Gmet_0936 of *Geobacter metallireducens* (Gme) HNH endonuclease was cloned, expressed, and purified following standard protocols developed by the Northeast Structural Genomics Consortium (NESG) for production of selenomethionine enriched protein sample [7]. Briefly, the gene coding for Gme HNHE (NESG ID number: GmR87) was cloned into a pET21 (Novagen) derivative, yielding the plasmid pGmR87-21.1. The resulting construct contains eight non-native residues at the C-terminus (LEH_6_, H_6_ denoting a 6xHis-tag) that facilitate protein purification. *E. coli* BL21 (DE3) pMGK cells, a rare codon translation enhanced strain, were transformed with pGmR87-21.1, and cultured in MJ9 minimal medium supplemented with selenomethionine, lysine, phenylalanine, threonine, isoleucine, leucine, and valine for the production of selenomethionine-labeled HNHE [8], [9]. The cells were induced overnight at 17°C using 1 mM IPTG after culturing at 37°C to mid-log phase, and were harvested by centrifugation. Cell pellets were resuspended in a lysis buffer (50 mM Tris-HCl, pH 7.5, 500 mM NaCl, 40 mM imidazole, and 1 mM TCEP) and disrupted by sonication. The resulting lysate was clarified by centrifugation at 26,000×g for 45 min at 4°C. The supernatant was loaded onto a HisTrap HP column followed by a gel filtration column (Superdex 75 26/60, GE Healthcare). The purified Gmet_0936 protein was concentrated to ∼9 mg/ml. Sample purity (>98%) and molecular mass (13.3 kDa) were verified by SDS-PAGE and matrix-assisted laser desorption ionization time-of-flight mass spectrometry, respectively.

### Crystallization of Gmet_0936 Protein

Crystals of Gme HNHE were obtained by the hanging-drop vapor diffusion method. One to three µl of concentrated protein solution (9 mg/ml in 10 mM Tris-HCl, pH 7.5, 100 mM sodium chloride, 0.02% (w/v) sodium azide and 5 mM dithiothreitol) were mixed with 1 µl of the reservoir solution consisting of 0.2 M magnesium sulfate, 20% (v/v) polyethylene glycol 8000, and 100 mM Tris-HCl (pH 8.5), followed by incubation at 4°C for 1 week. The crystals were cryo-protected using paratone-N and flash-frozen in liquid propane for data collection.

### X-ray Diffraction Data Collection, Processing, and Structure Determination

Single anomalous dispersion (SAD) X-ray data were collected at wavelength 0.979 Å from a single crystal of the Gmet_0936 protein at 100 K on beam line X4A at the National Synchrotron Light Source at Brookhaven National Laboratory. The diffraction images were processed with the program package HKL2000 [10]. The positions of Se atoms and initial SAD phases were determined with program BnP [11]. These phases were used by program Phenix [12] for automatic model building. Three Se atoms are correlated to position of Met52 in the three molecules. Another three Se atoms found by BnP turned out to be Zn atoms in the structure, one bound to each protein molecule. Manual model rebuilding was carried out with COOT [13]. The atomic models were refined using the program Phenix [14], with translation, libration, and screw-rotation (TLS) parameters. Final model has R_free_ of 0.266 and standard R factor of 0.206 (Table 1). The Ramachandran plot showed that 92.7% residues are located in the most favored regions, and 7.3% are in additional allowed regions.

**Table 1:** Summary of crystallographic information.

Resolution range (Å)	30-2.6
Number of observations	613,776
*R*_merge_ (%)^1^	8.2 (46.8)
I/σI	29.0 (4.9)
Redundancy	10.6 (10.0)
Completeness (%)	98.7 (95.1)
*R* factor^2^ (%)	20.5 (23.7)
free *R* factor^2^ (%)	26.6 (31.0)
rms deviation in bond lengths (Å)	0.008
rms deviation in bond angles (°)	1.1
PDB entry codes^2^	4H9D

1 The numbers in parentheses are for the highest resolution shell, 2.7-2.6 Å for data processing statistics and 2.86-2.6 Å for refinement statistics.

2 The coordinates and structure factors have been deposited in PDB and are accessible under accession number 4H9D.

### Expression of Gmet_0936 From the IMPACT Protein Expression System

A synthetic gene coding for Gmet_0936 in pIDTSMART-Kan (IDT) with optimized *E. coli* codons was subcloned into a T7 expression vector pET21b (the insert was flanked by NdeI and XhoI sites). The C-terminal 6xHis-tagged protein, Gmet_0936 or its mutant variants were partially purified through a nickel spin column from 10–20 ml IPTG-induced cell culture. For medium-scale purification, 1 to 2 L of cells ER2566 [pET21b-gmet_0936] were grown at 37°C to late log phase and IPTG was added to a final concentration of 0.5 mM for protein induction at 30°C for 3 h. Gmet_0936 (H_6_) protein was purified through a nickel column by gravity flow using a protocol recommended by the manufacturer (nickel agarose FF, Qiagen). It was further purified by chromatography through a HiTrap Heparin HP column (5 ml, GE Healthcare). Gmet_0936 (H_6_) protein peak eluted at the NaCl concentration of 0.5 to 0.55 M. Alternatively, the NdeI-XhoI fragment was inserted into pTYB1 vector to be in fusion with intein and chitin-binding domain (CBD). The fusion protein Gmet_0936-intein-CBD was purified from a chitin column (NEB) and Gmet_0936 was cleaved off by DTT and eluted. Gmet_0936 was further purified by chromatography through a HiTrap Heparin HP column (5 ml, GE Healthcare), protein was eluted by a NaCl gradient of 50 mM to 1 M and eluted fractions were analyzed by SDS-PAGE. Purified fractions containing Gmet_0936 protein was stored in 0.25 M NaCl, 20 mM Tris-HCl, pH 7.5, 50% glycerol, 1 mM DTT at −20°C. Reaction buffer contains 50 mM NaCl, 10 mM Tris-HCl, pH 6 to 8, supplemented with divalent cations (Mg^2+^, Mn^2+^, Co^2+^, Ni^2+^, or Zn^2+^) as specified in each reaction. Digestion was carried out at 37°C for 1–2 h or overnight, and reaction was stopped by addition of a stop dye solution with EDTA (10 mM) and SDS (0.1%).

### DNA Mobility Shift Assay

FAM-labeled duplex oligos were incubated with 6xHis-tagged N77H or Gme HNHE at room temperature for 20 min and the DNA-protein complexes were resolved in a 10% PAG gel. The image of DNA-protein complex was derived by scanning on a variable mode imager (Typhoon 9400, GE Healthcare).

Oligo 1 sequence: 5′ ACG ATA GTT ACC GGA TAA GGC GCA GCG G 3′ (ACCGG-top).

Oligo 2 sequence: 5′ CCG CTG CGC CTT ATC CG G TAA CTA TCG T-**FAM** 3′ (ACCGG-bottom).

### Preparation of Acid-damaged Plasmid DNA (heat/acid Depurination of DNA)

The published procedure for acid and heat damaged (depurinated) DNA was followed [15]. Briefly, 20 µg of pBR322 DNA was treated in 20 mM citrate acetate (pH 5.0), 0.1 M NaCl, at 70°C for 1 to 2 h in a total reaction volume of 100 µl. The acid/heat treated DNA was then purified through a Qiagen spin column and resuspended in 10 mM Tris-HCl buffer (pH 8) and then used for nicking reactions with Gme HNHE or its variants.

### Site-directed Mutagenesis of Gmet_0936 to Create Variants D53A, H54A, N68A, and N77H

Gmet_0936 variants were created by PCR mutagenesis using a pair of non-overlapping primers and Phusion DNA polymerase® (NEB). PCR products were purified through spin columns, phosphorylated by T4 polynucleotide kinase and ligated by T4 DNA ligase. Ligated DNA was used to transform *E. coli* competent cells ER2566 and transformants were selected on LB agar Amp plates. The desired mutations were confirmed by DNA sequencing of the alleles on plasmids.

### Accession Numbers

The PDB entry codes (coordinates and structure factors) have been deposited in pdb database and received the accession number: 4H9D.

## Results and Discussion

### Identification of Gmet_0936 Protein Homologs

**Figure 1:**
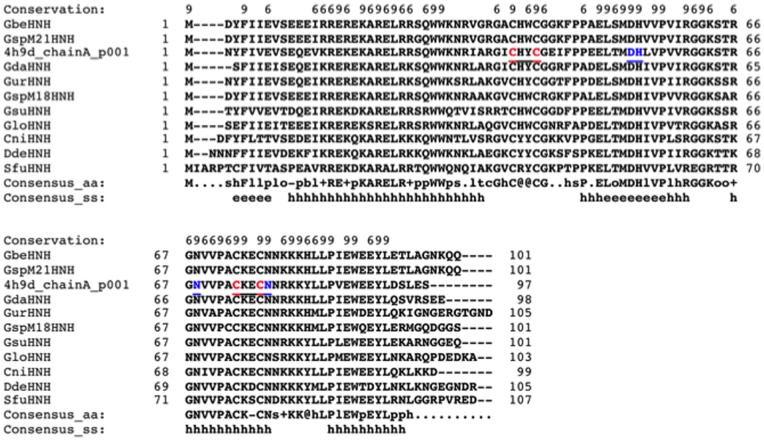
PROMALS3D multiple sequence alignment of putative HNH endonucleases. Consensus predicted secondary structure symbols: alpha-helix: h; beta-strand: e. Consensus amino acid symbols are: conserved amino acids are in bold and uppercase letters; aliphatic (I, V, L): l; aromatic (Y, H, W, F): @; hydrophobic (W, F, Y, M, L, I, V, A, C, T, H): h; alcohol (S, T): o; polar residues (D, E, H, K, N, Q, R, S, T): p; tiny (A, G, C, S): t; small (A, G, C, S, V, N, D, T, P): s; bulky residues (E, F, I, K, L, M, Q, R, W, Y): b; positively charged (K, R, H): +; negatively charged (D, E): −; charged (D, E, K, R, H): c. 4 h9d_chainA, Gme HNHE from *G. metallireducens* GS-15 (15 C-terminal residues EPSDGEGLEH_6_ are disordered and not resolved in the structure. Other HNH endonuclease homologs in the alignment are: Gbe, *G. bemidjiensis* Bem; GspM21, *Geobacter* sp. M21; Gda, *G. daltonii* FRC-32; Gur, *G. uraniireducens* Rf4; GspM18, *Geobacter* sp. M18; Gsu, *G. sulfurreducens* PCA; Glo, *G. lovleyi* SZ; Cni, *Calditerrivibrio nitroreducens* DSM 19672; Dde, *Deferribacter desulfuricans* SSM1; Sfu, *Syntrophobacter fumaroxidans* MPOB.

A BlastP search for homologous proteins in GenBank using Gmet_0936 as a query identified over 50 small putative HNH endonucleases (92 to 133 aa in length) with more than 50% amino acid (aa) sequence identity. The top ten close homologs with the predicted secondary structure are shown in **Fig. 1** (PROMAL3D alignment, the rest of homologs not shown) [16]. Gmet_0936 homologs can be found in *Geobacter* species and some members of Geobacteraceae, Desulfuromonadales, and Pelobacteraceae. For example, Gmet_0936 shares 75% aa sequence identity to a putative HNHE (protein ID: YP_001231596) from *Geobacter uraniireducens*. It also shares 76% aa sequence identity to a putative HNHE (protein ID: NP_953119) encoded in the genome of *Geobacter sulfurreducens* PCA strain. The presence of a large number of close homologs suggests that Gmet_0936 and its relatives may have some unknown biological function, possibly in DNA repair or recombination.

### Crystal Structure of the Putative HNH Endonuclease

**Figure 2:**
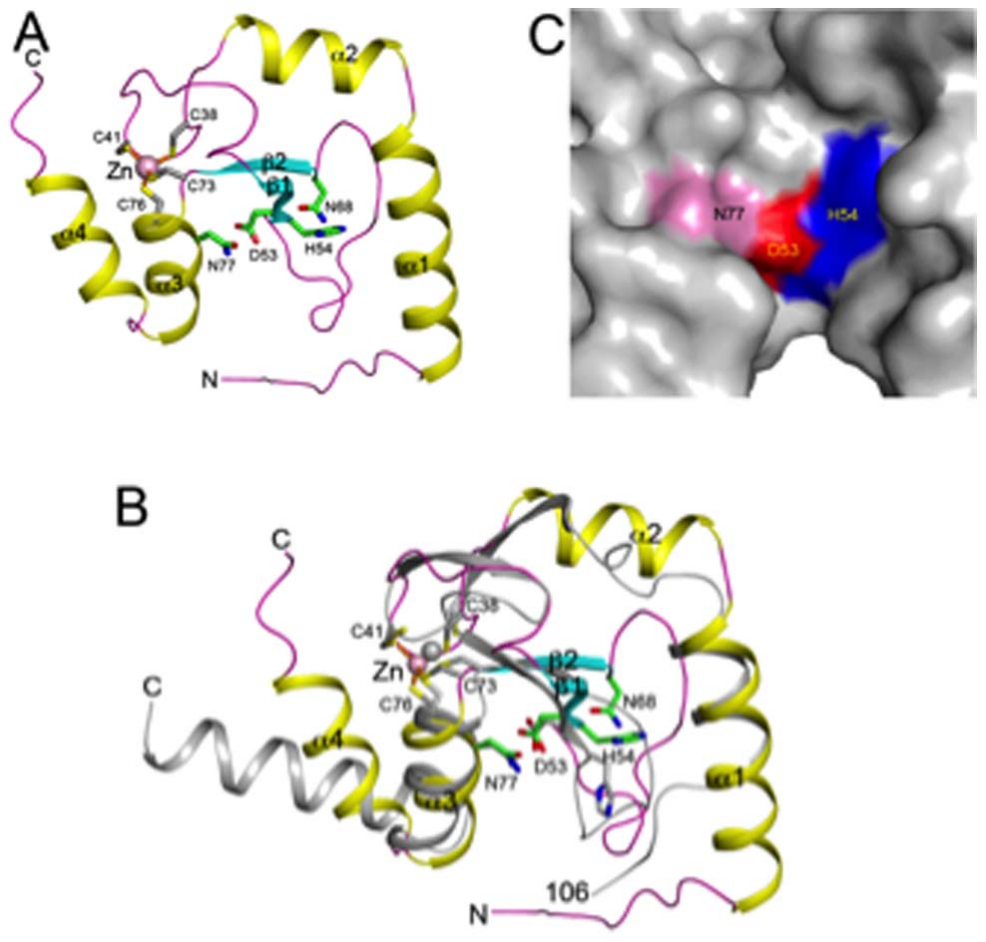
Crystal structure of the Gmet_0936 protein, a putative HNH endonuclease from *Geobacter metallireducens* GS-15. **A.** Schematic drawing of the structure of Gmet_0936 protein. The secondary structure elements are labeled. A bound zinc ion and its four Cys ligands are shown as a sphere (pink) and in stick models (in gray), respectively. Residues 53, 54, 68, 77 are shown as stick models (in green). **B.** Overlay of the structure of HNH endonuclease (in color) with the restriction endonuclease Hpy99I (in gray) [18]. Residues in the zinc binding site and the active site are shown as stick models. The N-terminal residues of Hpy99I (1–105) are not shown. **C.** Molecular surface of the active site region of the HNH endonuclease. Residues His54, Asp53 and Asn77 are given different colors and labeled.

Crystal structure of Gmet_0936 protein, a putative HNH endonuclease from *G. metallireducens* GS-15 was determined at 2.6**Å resolution, using selenomethionyl (Se-Met) derivatized protein and single-wavelength anomalous dispersion method of phase determination. The structure model was refined to working and free R-factors of 0.206 and 0.266, respectively. The data processing and refinement statistics are presented in Table 1. There are 3 molecules of the Gmet_0936 protein in the asymmetric unit, named A, B, and C, containing residues 1–98, 5–95, and 16–95 respectively. The structure of each monomer contains a two-stranded anti-parallel β-sheet (β1–β2) that is surrounded by two helices on each face (α1–α4) (**Fig. 2A**). The overall shape of the monomer is rather flat, with dimensions of 35×30×15 Å^3^. The conformations of the three molecules in the asymmetric unit are similar, with rms distance of ∼0.7 Å among their equivalent Cα atoms. The structure revealed a Zn ion associated with each monomer, coordinated by residues Cys38, Cys41 (in the long loop connecting helix α2 and strand β1), Cys73, and Cys76 (near the N-terminal end of helix α3) (**Fig. 2A**). The zinc ion likely plays an important structural role, stabilizing the overall conformation of the Gmet_0936 protein monomer.

The Gme endonuclease belongs to the family of HNH endonucleases which is also known as ββα-Me endonucleases. This conserved ββα Me fold with a bound zinc ion has been found in a wide range of nucleic acid enzymes including non-specific endonucleases, type II restriction enzymes, homing endonucleases, holiday junction resolvase, and DNA repair enzymes. We next performed homolog search of known structures in pdb. Structural homologs of the Gme HNHE was identified with the program DaliLite [17]. These include the restriction endonuclease Hpy99I (CGWCG, PDB code 3FC3) [18], phage T4 endonuclease VII, a holiday junction specific endonuclease (PDB code 2QNC) [19], periplasmic nuclease Vvn from *Vibrio vulnificus* (1OUO) [20], and endonuclease I from bacterium *Vibrio salmonicida* (2PU3) [21], with *Z* scores of 4.3, 3.9, 3.5, and 3.5, respectively. The root mean squared distance for ∼65 equivalent Cα atoms is 3.1–3.6 Å, and the proteins share 15–20% amino acid sequence identity with Gme HNHE. An overlay of the structures of Gme HNHE and Hpy99I is shown in **Fig. 2B**. Overall, most of the secondary structure elements, as well as the zinc binding site, have counterparts in the two protein structures. Moreover, the catalytic residues in the active site are also mostly conserved between the two structures (see below). On the other hand, Hpy99I contains extra structural elements at the N-terminus (residues 1–105), which mediate the binding of the DNA substrate as well as dimerization [18]. These elements are absent in Gme HNHE (**Fig. 2B**). The lack of a companion DNA methyltransferase (MTase) and the ability of high expression of Gme HNHE protein in *E. coli*, and the lack of apparent DNA digestion pattern in Mg^++^ buffer led to the conclusion that Gme HNHE is not a typical type II restriction enzyme (see below for DNA nicking activity).

Sequence analysis suggests that residues Asp53, His54, Asn68 and Asn77 are in the active site of the HNH endonuclease. Residues Asp53 and His54 are at the end of strand β1, on the surface of the monomer, and Asn68 and Asn77 are located close to these two residues (**Fig. 2A**). Moreover, residues Asp53-His54 are equivalent to important catalytic residues (Asp148-His149) in the structural homolog Hpy99I (**Fig. 2B**), a ββα-Me type II REase [18]. Asn77 is equivalent to Asn165, although Asn68 does not have a counterpart in Hpy99I (**Fig. 2B**). Asn77 directly follows a ligand to the zinc ion (Cys76) in both Gme HNH endonuclease and Hpy99I, indicating a tight coupling between zinc binding and the organization of the active site. Overall, structural and sequence analyses have identified the putative active site of this HNH endonuclease (see mutagenesis data below). The conserved ββα-Metal fold is also found in the PacI restriction enzyme where the typical His residue is now occupied by an Arg residue in the catalytic site consisting of H_42_-R_93_-Y_100_-N_113_, further illustrating the diversity of the HNH family enzymes [22].

**Figure 3:**
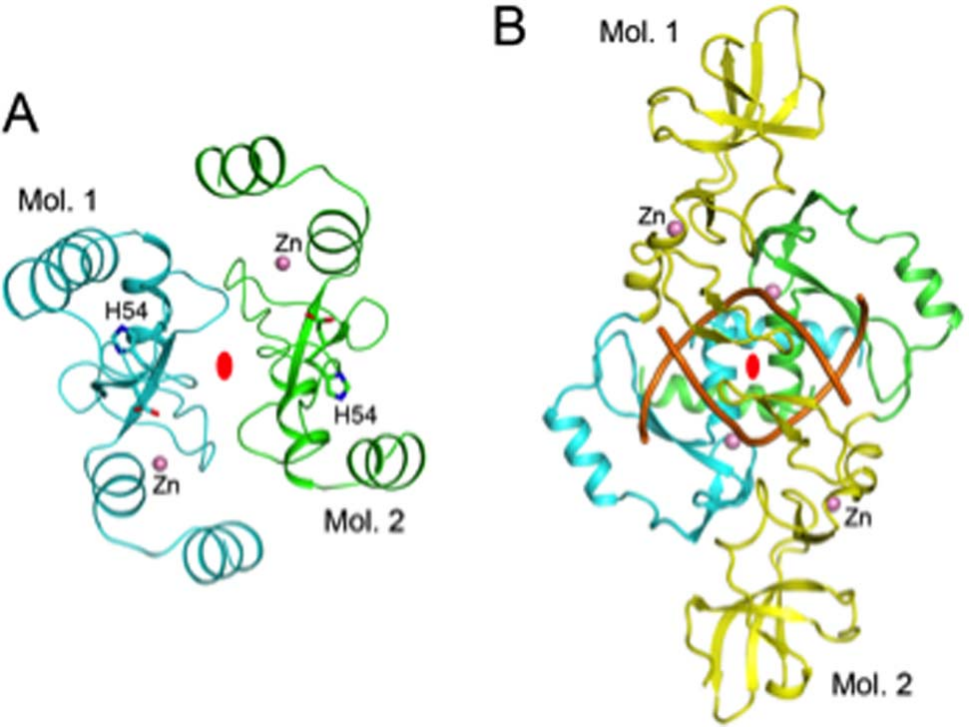
Structure of the Gmet_0936 protein dimer. **A.** Schematic drawing of the Gmet_0936 protein dimer. The two molecules are colored in cyan and green. Residues Asp53-His54 in the putative active site are shown as stick models. The two-fold axis of the dimer is indicated by the red oval. **B.** Structure of Hpy99I dimer in complex with duplex DNA [18]. The catalytic domains are colored in cyan and green, and the N-terminal segment in yellow. The DNA duplex is in orange.

**Figure 4:**
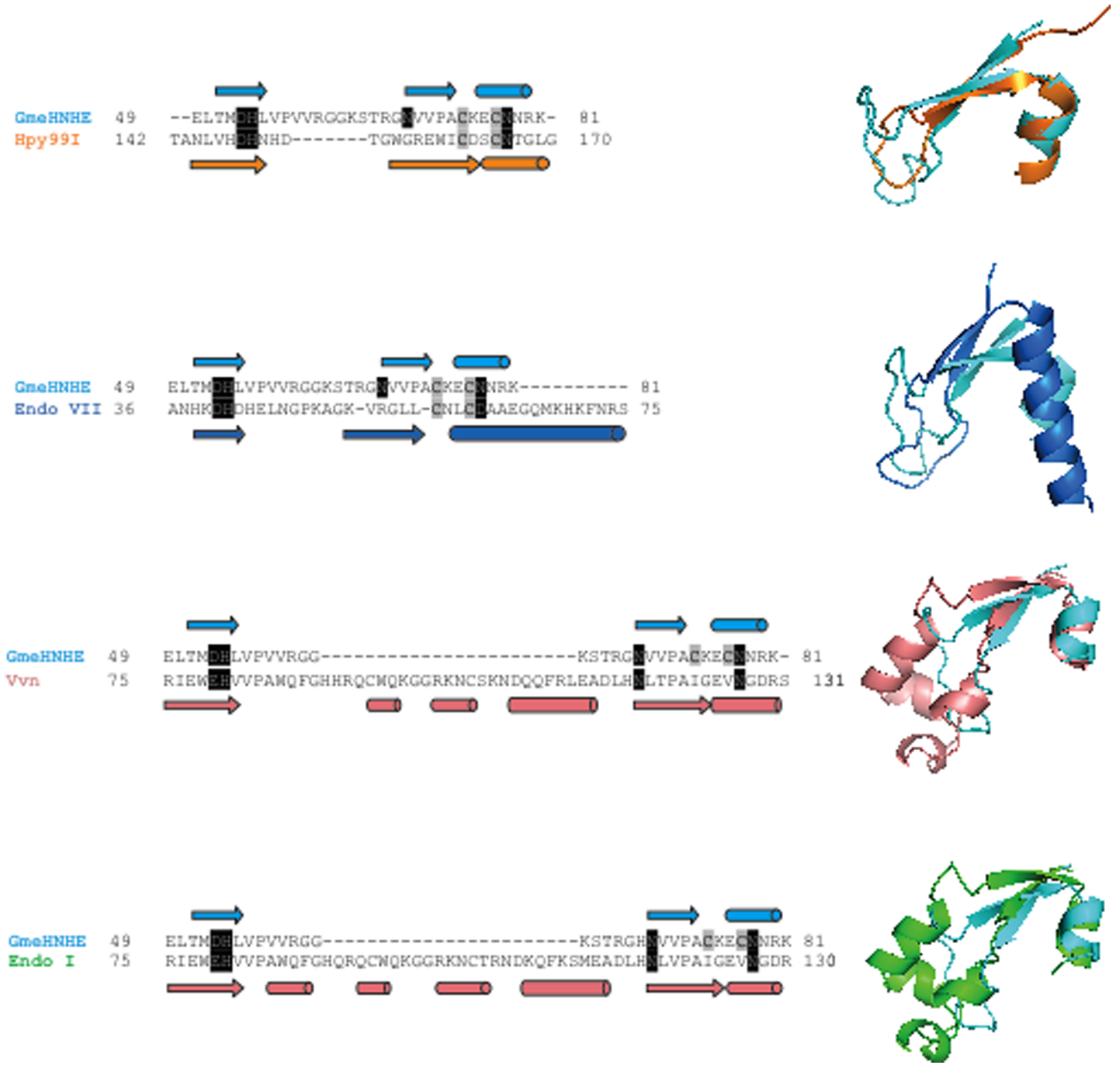
Structural comparison of the conserved ββα-metal fold and catalytic sites of Gme HNHE, Hpy99I, phage T4 endonuclease VII, periplasmic nuclease Vvn from *Vibrio vulnificus*, and endonuclease I from bacterium *Vibrio salmonicida*. Some additional secondary structures (β sheets) were inserted in the ββα-Me fold in Vvn nuclease and endonuclease I.

Studies in solution indicate that the Gme HNHE is primarily a dimer (see molecular sizing chromatography results below). Two of the three molecules in the crystallographic asymmetric unit form a dimer (**Fig. 3A**), which may correspond to the dimer in solution. Part of the interface of this dimer is ionic/hydrophilic in nature, including residues Arg32, Arg35, His39 and Arg66. However, the third molecule does not form a similar dimer. In fact, it appears to be mostly monomeric, and has only weak association with its crystallographic symmetry-mate. The crystallization solution, with 0.2 M Mg sulfate and therefore relatively high ionic strength, may have partly destabilized the dimer. The dimer of this nuclease is entirely different from that of Hpy99I, in complex with duplex DNA (**Fig. 3B**) [18]. Therefore, the binding mode of the Gme HNHE to its DNA substrate may be different as well. The HNH catalytic domain (ββα-metal fold) and catalytic residues are conserved among non-specific endonucleases, HNH-family restriction enzymes, and DNA repair endonuclease such as phage T4 endonuclease VII (DNA resolvase to release arrested Y-DNA structure). **Fig. 4** shows the catalytic residues of Gme HNHE, Hpy99I, phage T4 endonuclease VII, Vvn nuclease, and endonuclease I. A large group of HNH-family DNA nicking enzymes encoded by phage and prophage were discovered recently [23]. These nickases display low amino acid sequence similarity to Gme HNHE. Some type IV restriction enzymes such as Sco_McrA and *E. coli* McrA also have the conserved HNH catalytic domain and a zinc binding site. Sco_McrA cleaves phosphorothioated DNA as well as methylated DNA and prefers Mn^2+^ or Co^2+^ for catalytic activity [24]. Thus, the property of Mn^2+^ –dependent catalytic activity is shared by Gme HNHE, Sco_McrA endonuclease, and some phage/prophage encoded nicking enzymes. *E*. *coli* McrA restricted incoming 5 mC modified λ phage or plasmid DNA or 5 hmC-modified T4gt [25] [26]. Although the type IV restriction enzyme McrA (or McrA homologs) and phage-encoded HNH nicking enzymes share low amino acid sequence similarity to Gmet_0936, the structure-guided sequence alignment (Phyre server) [27] shows that these enzymes display similar predicted structure models in the zinc binding site and the HNH (HNN) catalytic domain (data not shown).

The Zn binding site of Gme HNHE is reminiscent of the zinc ribbon of the 134-aa nuclease domain of Colicin E9 containing a zinc-finger-like HNH motif that binds divalent transition metal ions (Zn^2+^, Co^2+^, or Ni^2+^) with binding affinity for Zn^2+^ three order of magnitude higher than other divalent cations. The metal ion is coordinated by three His residues and Zn^2+^ is not required for the endonuclease activity, but necessary for structure stabilization [28].

### Purification of Gmet_0936 Protein and its N77H Variant and DNA Binding/nicking Assays

The 6xHis-tagged (13.3 kDa) and none-His-tagged Gmet_0936 protein (12.7 kDa) purified from chitin/Heparin columns showed slightly larger apparent molecular size than the predicted molecular masses (**Fig. S1A**). By multiple sequence alignment with other known HNH endonucleases and by structure-guided analysis, the possible catalytic residues of Gmet_0936 protein are D53, H54, N68, and N77. N77 position is usually occupied by a His residue in many HNH endonucleases (gp74 endonuclease of phage HK97 also has a His residue immediately after the CxxC motif) [29]. Therefore, N77 was mutated to His by site-directed mutagenesis and the His-tagged protein N77H (H_6_) was partially purified (**Fig. S1B and C**) and assayed for endonuclease activity.

**Figure 5:**
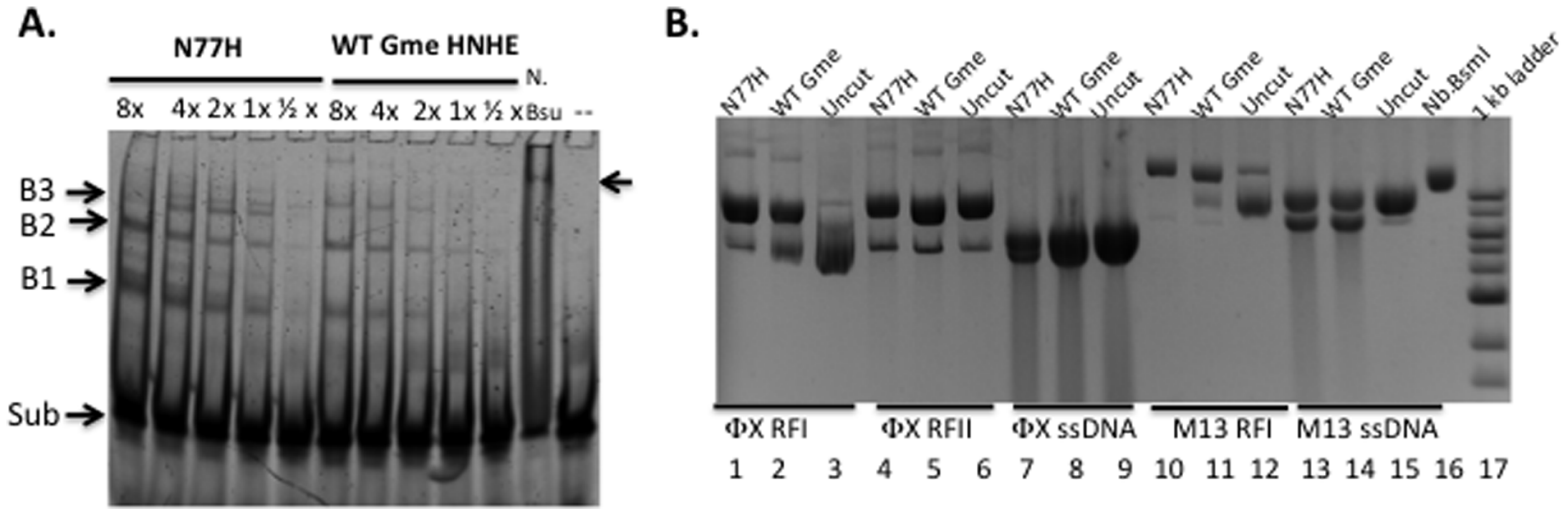
DNA mobility shift assay (DNA binding assay) in the absence of divalent cations and DNA nicking activity assays for 6xHis-tagged N77H in buffers with different metal ions. **A.** Fluorescein-labeled duplex oligos were incubated with varying amount of 6xHis-tagged N77H or Gme HNHE at room temperature for 20 min and the DNA-protein complexes were resolved in a 10% PAG gel. ½ to 8× indicates the relative protein concentration (1x = 0.5 µg protein). Arrows indicate the substrates and the bound complexes. N.Bsu, *Bacillus subtilis* HNH nicking endonuclease. The binding buffer in this assay lacks divalent cations and therefore does not support DNA nicking. **B.** DNA nicking assay for WT and N77H (H_6_). Lanes 1–3, φX174 RFI DNA (dsDNA); lanes 4–6, φX174 RFII DNA (dsDNA, nicked circular); lanes 7–9, φX174 ssDNA; lanes 10–12, M13 RFI (dsDNA); lanes 13–15, M13 ssDNA; lane 16, nicked M13 RFI DNA by Nb.BsmI; lane 17, 2-log DNA ladder.

**Fig. 5A** shows that at 20-fold molar excess of protein vs DNA, 2–3 protein bound complexes were detected in the gel shift assay. At higher protein concentration, more substrate DNA was bound and shifted towards slow migrating complexes. Since the precise binding specificity of the Gmet_0936 (H_6_) and its N77H (H_6_) variant is still unknown, the bound complexes are probably resulted from non-specific DNA binding activity. In the control experiment, the Bsu HNH endonuclease which has a DNA nicking specificity of CG↓GT (ACCG) bound the duplex oligos and formed a high molecular weight complex (**Fig. 5A**, lane 11). It was concluded that Gmet_0936 (H_6_) and N77H (H_6_) variant are weak DNA binding proteins. Gme HNHE may need other DNA binding partner(s) for high affinity DNA binding.

We next digested various DNA substrates with purified Gme HNHE and N77H (H_6_) enzymes in buffers with different divalent cations such as Mg^2+^, Mn^2+^, Zn^2+^, Ca^2+^, Co^2+^, or Ni^2+^ at various pH levels (pH 6 to 8). N77H (H_6_) enzyme displays detectable DNA nicking activity in Mn^2+^ buffers at low pH (pH 6 to 7) (data not shown). Purified Gme HNHE and N77H (H_6_) enzymes display weak DNA nicking activity in overnight digestions at pH 7 in a Mn^++^ buffer. **Fig. 5B** (lanes 1–2, 10–11) shows that N77H (H_6_) and Gme HNHE enzymes nick dsDNA (φX174 and M13 RFI DNA) and they also changed the migration pattern of ssDNA (M13 ssDNA, lanes13–15) in the overnight digestion. Both enzymes do not appear to cleave pre-nicked φX174 RFII DNA (lanes 4–6). Multiple attempts to determine the nicking specificity (nicking site) of N77H (H_6_) were not successful when nicked and gel-purified pBR322 DNA was used as templates for run-off sequencing, suggesting that N77H (H_6_) endonuclease nicks plasmid DNA non-specifically. In a control experiment, nicked pBR322 by site-specific and strand-specific nicking enzymes (N.φGamma) gave rise to sharp drop in sequencing peaks in run-off sequencing (data not shown).

### N77H (H_6_) Endonuclease Prefers Nicking on Acid and Heat Treated DNA

Since Gme N77H (H_6_) endonuclease is more active in low pH buffers (activity in pH 6, 6.5, and 7) it was suspected that the rate of DNA depurination was enhanced at the lower pH levels (pH 6–7), resulting in the apparent elevated DNA nicking activity. Therefore we tested the nicking activity of Gme N77H (H_6_) enzyme on acid and heat damaged pBR322 DNA. **Fig. S2A** shows the partial nicking activity of N77H (H_6_) on regular pBR322 in an Mn^++^ buffer at pH 7.0. **Fig. S2B** shows that following acid and heat treatment for 2 h, some supercoiled DNA was converted to nicked circular DNA (breaks of DNA backbones in addition to depurination) (see lane 1, zero time point). Most of the supercoiled DNA was converted to nicked circular DNA after 90 min digestion by N77H (H_6_). It was concluded that the DNA nicking activity of N77H (H_6_) could be enhanced by acid and heat treatment of plasmid DNA. However, we cannot exclude other catalytic activity for this enzyme, which may be more physiologically relevant.

### Site-directed Mutagenesis of the Putative Active site

We also constructed four other 6xHis-tagged variants: D53A, H54A, N68A, and N77A; and these four mutant proteins were partially purified from nickel spin columns. The protein expression level in crude cell lysate and in nickel column purified fractions was normal for the D53A variant, but much reduced for the H54A and N68A variants (∼5 to 10% of wt), and poorly expressed for the K14E/N77A double mutant (we were unable to isolate N77A single mutant since Asn77 to Ala substitution was accompanied by a secondary mutation K14E) (**Fig. S3**). In addition, K14E/N77A transformation into a T7 expression strain (ER2566) was only about 0.4% of the wt Gme HNHE (data not shown), indicating that K14E/N77A may be toxic to the expression host, possibly due to its tight DNA binding and interference with transcription or DNA replication (it was noted that N77H plasmid was able to be transferred into the T7 expression strain at equal efficiency as the wt gene on the same vector). The Gme H54A variant is also toxic to *E. coli* with reduced transformation efficiency. The partially purified mutant proteins D53A, H54A, N68A displayed reduced nicking activity on pBR322 (buffer pH 7.0, 1 mM Mn^++^) (data not shown). The activity of K14E/N77A cannot be determined due to the poor expression and lack of purified protein. From the mutagenesis result, it was concluded that H54, N68, and N77 are critical for both enzyme activity and for maintaining proper structural folding as well. The role of D53 residue is less important for structure integrity and it’s negative charge of side chain may contribute to the catalytic site by stabilizing a metal ion.

### Gme HNHE is not a Holiday Junction Resolvase by Itself

Due to the structure similarity in the HNH catalytic domain of Gme HNHE and phage T4 endonuclease VII, we tested N77H (H_6_) variant in nicking pUC19 DNA and a pUC-derivative that contains a long stretch of [AT]_20_ tracks that can form a cruciform structure. Both plasmids were nicked non-specifically by N77H (H_6_) endonuclease, with equal nicking activity on the plasmid with a cruciform structure (data not shown). We concluded that Gme HNHE is unlikely a holiday junction resolvase by itself.

### Molecular Sizing Column Chromatography of non-His-tagged Gme HNHE

To determine the oligomerization state of Gme HNHE, we performed molecular sizing column chromatography for the enzyme (purified by chitin and Heparin columns). Ten µl of the purified non-His-tagged Gme HNHE (4 or 8 mg/ml) was injected into a Superdex 200 5/150 GL column (3 ml bed volume; GE Healthcare). The partition coefficient (K_av_) of Gmet_0936 protein was determined by K_av_ = (v_e_−v_o_)/(v_t_−v_o_) where v_e_ is the elution volume, v_o_ is the void volume of the column and v_t_ is the total column volume. v_o_ was determined empirically from the elution time of blue dextran and v_t_ was the total column volume 3 ml. A standard curve was created by obtaining the K_av_ values of the standard protein ribonuclease A (13.5 kDa), ovalbumin (43 kDa) and conalbumin (75 kDa) (GE Healthcare) run under the same conditions. The K_av_ values for two enzyme concentrations and the standard proteins are represented by open circles and block squares, respectively. The K_av_ values and the derived apparent molecular weight of Gmet_0936 protein are 0.899 and 0.891, corresponding to 26.2 kDa and 25.3 kDa for the 4 mg/ml and 8 mg/ml injection, respectively (**Fig. S4**). The apparent molecular weight of the Gme HNHE is consistent with a dimer configuration.

### Analytical Gel Filtration and Static Light Scattering of 6xHis-tagged Gme HNHE

To analyze the oligomerization state of the Gme HNHE with a C-terminal 6xHis tag (LEH_6_, the same protein in the crystal structure), Gme HNHE (H_6_) was purified to near homogeneity from a nickel column and a gel filtration column. Thawed aliquot of the protein crystallization stock was subjected to analytical gel filtration on a Shodex 802.5 column (Showa Denko, Tokyo, Japan) running at 4°C in 100 mM NaCl, 0.025% (w/v) NaN_3_, 100 mM Tris-HCl (pH 7.5). The column eluant was monitored using static light scattering and refractive index detectors (miniDAWN™ TREOS and Optilab T-rEX, Wyatt Technologies, Santa Barbara, CA). Debye analysis shows a molecular mass of 23.9 kDa, indicating that Gme HNHE (H_6_) is a dimer (data not shown), which is consistent to the result derived from molecular sizing column described above for the non-His tagged protein. In term of cleavage/nicking mechanism, it is not clear why Gme HNHE only nicks DNA and does not cause extensive ds breaks on acid/heat damaged DNA.

The biological function of Gme HNHE is unknown. Due to its weak DNA binding activity, Gme HNHE may require other protein partners to be recruited to DNA lesion sites, and introduce nicks at or near AP sites. With the availability of the 6xHis-tagged Gme HNHE and N77H variant, it is now possible to identify the protein interaction partner from cell extracts of *Geobacter metallireducens* GS-15 by binding the protein complex in a nickel agarose column, or using Gme HNHE-intein-CBD fusion to bind the interacting partner in co-purification from a chitin column. The N77H (H_6_) variant shows improved non-specific DNA nicking activity on acid and heat damaged plasmid DNA. Therefore, we suspect that Gme HNHE may play a role in DNA repair and/or recombination. The structural similarity of Gme HNHE and the C-terminus of Hpy99I restriction enzyme suggested divergent evolution of the HNH (HNN/HNK) family endonucleases: one towards possible DNA repair funtion (nicking of damaged DNA for homologous recombination repair) and the other towards DNA restriction by fusion with a sequence-specific DNA recognition domain. The structure of Gme HNHE reported here complements the structural information derived from other HNH family nucleases including His-Cys box homing endonuclease, restriction endonuclease, DNA repair enzyme, holiday junction resolvase, and non-specific Colicin endonucleases.

Recently, a protein called HlpB essential for cell viability with the conserved HNH motif was discovered in *Bacillus subtilis* (hlpB or yisB gene) [30]. The depletion of HlpB leads to growth arrest and to the generation of cells containing a single, decondensed nucleoid. Purified HlpB protein showed cooperative binding to ds and ssDNA in the presence of divalent cations, but no apparent DNA cleavage or nicking were detected. Lethality of the hlpB deletion was relieved by the knock out of addA and of addAB, two genes encoding proteins forming a RecBCD-like end resection complex. These results suggest that HlpB plays a role in DNA repair by rescuing AddAB-mediated recombination intermediates in *B. subtilis* and possibly has similar function in other bacteria. The HlpB (100 aa residues) and Gme HNHE (104 aa residues) only share 11% aa sequence identity. Based on the biochemical properties of HlpB and Gme HNHE, we suspect that Gme HNHE likely plays a different DNA repair function from that of HlpB protein. More genetic and biochemical studies are needed to pinpoint the biological function of Gme HNHE.

## Conclusion

The crystal structure of Gmet_0936 protein from *Geobacter metallireducens* GS-15 was determined at 2.6 Å resolution. The structure contains a two-stranded anti-parallel β-sheet surrounded by two helices on each face, and reveals a Zn binding site in each monomer, coordinated by residues Cys38, Cys41, Cys73, and Cys76. Structural homologs of Gmet_0936 include Hpy99I endonuclease, phage T4 endonuclease VII, and other HNH endonucleases with the conserved ββα-Me structural fold. An overlay of Gmet_0936 and Hpy99I structures shows that most of the secondary structure elements, catalytic residues as well as the zinc binding site (zinc ribbon) are conserved. However, Gmet_0936 lacks the N-terminal domain of Hpy99I, which mediates DNA binding as well as dimerization. Purified Gmet_0936 forms dimers in solution and a dimer of the protein is observed in the crystal, but with a different mode of dimerization as compared to Hpy99I. Gmet_0936 and its N77H variant show a weak DNA binding activity in a DNA mobility shift assay and a weak Mn^2+^-dependent nicking activity on supercoiled plasmids in low pH buffers. The preferred substrate is acid and heat-treated DNA with AP sites, suggesting that Gmet_0936 may be a DNA repair enzyme. Site-directed mutagenesis indicated that the catalytic residues His53, N68, and N77 are also important for maintaining proper structure folding of the protein.

## Supporting Information

Figure S1 **SDS-PAGE analysis of purified His-tagged and non-His-tagged proteins. A.** Partially purified proteins: 6xHis-tagged Gme HNHE with the non-native C-terminal amino acid (aa) residues LEH_6_, predicted molecular mass: 13,264 Dalton, pI = 6.71. Gme HNHE with non-native C-terminal residues LEGSS following intein cleavage, predicted molecular mass: 12,672 Dalton, pI = 5.43. Lane 1, IPTG-induced and clarified cell lysate (SN, supernatant); lanes 2 and 3, flow-through (FT) from the nickel column; lanes 4 and 5, eluted fractions containing Gme HNHE (H_6_) protein; lanes 6 and 8, eluted fractions containing Gme HNHE from a Heparin column purification; lane 7, chitin column eluent containing Gme HNHE after DTT cleavage; lane 9, protein ladder (NEB). **B.** SDS-PAGE analysis of partially purified N77H (H_6_) protein. Lane 1, eluent from a nickel column; lane 2, IPTG-induced and clarified cell lysate (SN, supernatant); lane 3, total proteins in cell lysate; lane 4, protein ladder. **C.** SDS-PAGE analysis of purified N77H (H_6_) protein eluted fractions from a Heparin column.
(TIFF)

Figure S2 **N77H (H_6_) DNA nicking activity assays on regular pBR322 and acid/heat damaged pBR322 DNA. A.** A time course of N77H (H_6_) nicking activity on pBR322. PstI, linear DNA; Nt, nicked circular form by Nt.BspQI; nicking reaction time course (10 to 90 min) at 37^o^C in an Mn^++^ buffer (1 mM). zero = DNA plus buffer, no enzyme added. **B.** A time course of N77H (H_6_) nicking activity on acid/heat damaged pBR322 (sodium citrate, pH 5.0, 70^o^C for 2 h pre-treatment to inflict damage). Following acid/heat treatment, a fraction of the supercoiled DNA had been converted to linear and nicked circular forms (see zero time point).
(TIFF)

Figure S3 **SDS-PAGE analysis of Gme HNHE variants in cell extract and in partially purified protein fractions from nickel spin columns. A.** SDS-PAGE analysis of WT Gme HNHE and its variants. Lane 1–2, protein ladder and purified Gme HNHE; lanes 3–9, IPTG-induced cell extracts containing WT Gme HNHE, and its variants D53A, H54A, N68A, N77H, and K14E/N77A (two isolates). **B.** SDS-PAGE analysis of partially purified WT Gme HNHE and its variants. Lane 10–11, protein ladder and purified WT Gme HNHE (Heparin); lanes 12–18, partially purified enzymes from nickel spin columns: WT Gme HNHE and its variants D53A, H54A, N68A, N77H, and K14E/N77A (two isolates).
(TIFF)

Figure S4 **Molecular sizing column chromatography to determine the oligomerization state of the Gme HNHE.** The partition coefficient (K_av_) of Gme HNHE was determined by K_av_ = (v_e_−v_o_)/(v_t_−v_o_) where v_e_ is the elution volume, v_o_ is the void volume of the column and v_t_ is the total column volume. v_o_ was determined empirically from the elution time of blue dextran and v_t_ was the total column volume 3 ml. A standard curve was created by obtaining the K_av_ values of the standard protein ribonuclease A (13.5 kDa), ovalbumin (43 kDa) and conalbumin (75 kDa) run under the same conditions. The K_av_ values for two enzyme concentrations and the standard proteins are represented by open circles and block squares, respectively. The K_av_ values and the derived apparent molecular weight of Gme HNHE are 0.899 and 0.891, corresponding to 26.2 kDa and 25.3 kDa for the 4 mg/ml and 8 mg/ml injection, respectively.
(TIFF)
