# Supplementary figures and images for: Structure Determination and Biochemical Characterization of a Putative HNH Endonuclease from Geobacter metallireducens GS-15
Source: PLoS One. 2013 Sep 6;8(9):e72114. doi: 10.1371/journal.pone.0072114 (PMC3765158; doi:10.1371/journal.pone.0072114)

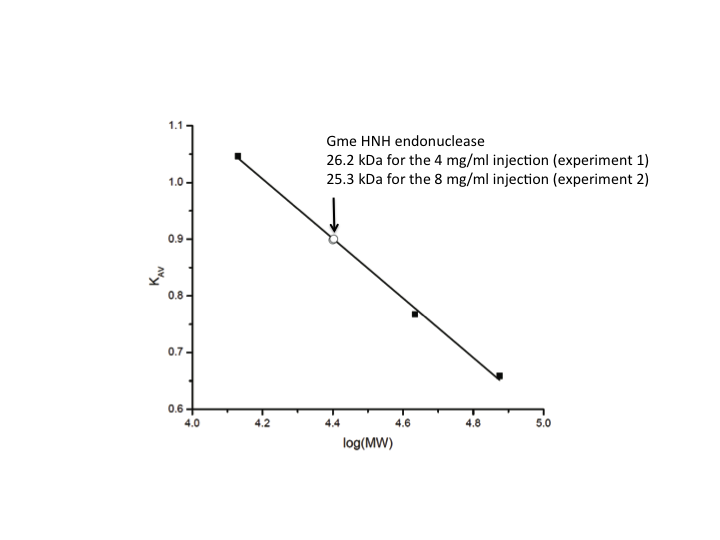

Supplement: Figure S4 — Molecular sizing column chromatography to determine the oligomerization state of the Gme HNHE. The partition coefficient (Kav) of Gme HNHE was determined by Kav = (ve−vo)/(vt−vo) where ve is the elution volume, vo is the void volume of the column and vt is the total column volume. vo was determined empirically from the elution time of blue dextran and vt was the total column volume 3 ml. A standard curve was created by obtaining the Kav values of the standard protein ribonuclease A (13.5 kDa), ovalbumin (43 kDa) and conalbumin (75 kDa) run under the same conditions. The Kav values for two enzyme concentrations and the standard proteins are represented by open circles and block squares, respectively. The Kav values and the derived apparent molecular weight of Gme HNHE are 0.899 and 0.891, corresponding to 26.2 kDa and 25.3 kDa for the 4 mg/ml and 8 mg/ml injection, respectively. (TIFF) [file pone.0072114.s004.tiff]
